# Global Metabolomics Discovers Two Novel Biomarkers in Pyridoxine-Dependent Epilepsy Caused by ALDH7A1 Deficiency

**DOI:** 10.3390/ijms232416061

**Published:** 2022-12-16

**Authors:** Hans-Otto Böhm, Mazyar Yazdani, Elise Mørk Sandås, Anja Østeby Vassli, Erle Kristensen, Helge Rootwelt, Hanne Bendiksen Skogvold, Eylert Brodtkorb, Katja Benedikte Prestø Elgstøen

**Affiliations:** 1 Department of Medical Biochemistry, Oslo University Hospital, Rikshospitalet, Sognsvannsveien 20, 0372 Oslo, Norway; 2 Department of Mechanical, Electronic and Chemical Engineering, Faculty of Technology, Art and Design, Oslo Metropolitan University, Pilestredet 35, 0166 Oslo, Norway; 3 Department of Neuroscience, Faculty of Medicine, Norwegian University of Science and Technology, 7491 Trondheim, Norway; 4 Department of Neurology, St. Olav’s Hospital, University Hospital, 7006 Trondheim, Norway

**Keywords:** pyridoxine-dependent epilepsy, *ALDH7A1*, UHPLC-HRMS, global metabolomics, biomarker, 6-hydroxy-(S)-2-aminocaproic acid (HACA), C_9_H_11_NO_4_ isomer

## Abstract

Pyridoxine-dependent epilepsy (PDE) is a rare autosomal recessive developmental and epileptic encephalopathy caused by pathogenic variants in the *ALDH7A1* gene (PDE-ALDH7A1), which mainly has its onset in neonates and infants. Early diagnosis and treatment are crucial to prevent severe neurological sequelae or death. Sensitive, specific, and stable biomarkers for diagnostic evaluations and follow-up examinations are essential to optimize outcomes. However, most of the known biomarkers for PDE lack these criteria. Additionally, there is little discussion regarding the interdependence of biomarkers in the PDE-ALDH7A1 metabolite profile. Therefore, the aim of this study was to understand the underlying mechanisms in PDE-ALDH7A1 and to discover new biomarkers in the plasma of patients using global metabolomics. Plasma samples from 9 patients with genetically confirmed PDE-ALDH7A1 and 22 carefully selected control individuals were analyzed by ultra high performance liquid chromatography–high-resolution mass spectrometry (UHPLC-HRMS). Two novel and reliable pyridoxine-independent diagnostic markers, 6-hydroxy-2-aminocaproic acid (HACA) and an isomer of C_9_H_11_NO_4_, were identified. Furthermore, a possible reaction mechanism is proposed for HACA. This study demonstrates the capability of global metabolomics in disease screening to detect established and novel biomarkers.

## 1. Introduction

Pyridoxine-dependent epilepsy is a group of autosomal recessively inherited metabolic diseases affecting the metabolism of pyridoxal 5′-phosphate (PLP). Pyridoxine-dependent epilepsy usually presents with neonatal epileptic encephalopathy that is resistant to treatment with conventional antiseizure medications but responsive to pyridoxine. PLP, the bioactive vitamer of vitamin B6, is a cofactor for a vast amount of enzymes involved in the function of mitochondria, the synthesis of proteins and polyamines, and the metabolism of carbohydrates, lipids, folate, amino acids, and neurotransmitters [1,2]. The underlying mechanism of PDE was initially attributed to a shortage of glutamic acid decarboxylase (GAD), which catalyzes the conversion of glutamate to gamma-aminobutyric acid (GABA) in the presence of pyridoxine [3,4]. In 2006, the discovery of biallelic pathogenic variants in the gene *ALDH7A1* (encodes alpha-aminoadipic semialdehyde dehydrogenase = ALDH7A1) revealed the key molecular basis of the disease [5].

ALDH7A1 is a downstream enzyme in the catabolism of lysine. ALDH7A1 deficiency leads to an accumulation of lysine degradation metabolites, some of which are believed to be neurotoxic, giving rise to the developmental delay that is seen in 75% of patients [6,7] despite early treatment with pyridoxine and seizure control. Consequently, adjunct lysine reduction therapy has recently been introduced as part of the treatment. Due to the neonatal or infantile onset in most cases, early diagnosis is essential to optimize clinical outcomes and thereby minimize the negative aspects of the disease on patients, families, and society. This underlines the importance of sensitive, specific, and stable disease markers for diagnostic evaluations and follow-up examinations. The current biomarkers for PDE-ALDH7A1 are alpha-aminoadipic semialdehyde (alpha-AASA), pipecolic acid Δ^1^-piperideine 6-carboxylate (P6C), pipecolic acid (PIP), 6-oxopiperidine-2-carboxylic acid (6-oxo PIP), 2S,6S- and 2S,6R-oxopropylpiperidine-2-carboxylic acid (2-OPP), and (2S, 6S/R)-6-(2-carboxymethyl)-piperidine-2-carboxylic acid (2-CMP). Unfortunately, most of these biomarkers suffer from several drawbacks such as a lack of stability (e.g., alpha-AASA and P6C), specificity (e.g., PIP), or well-defined origin (e.g., 6-oxo PIP, 2-OPP, and 2-CMP) [8,9,10,11].

To date, most research on PDE-ALDH7A1 metabolites has tended to focus on known biochemical changes and metabolite alterations [2]. Such hypothesis-testing approaches may provide analyses of a small number of specific metabolites but fall short of presenting the whole picture [12]. It is particularly challenging when differences between two study groups (e.g., case and control) are largely unknown or the cause of the disease in a patient is unclear [13,14]. This can now be resolved through high-resolution mass spectrometry (MS)-based global metabolomics. While a chemical name cannot initially be assigned to the majority of the features that are detected, an analysis of those that are identified allows for a biological interpretation by differential analysis and biochemical pathway mapping [15]. With the gradual shift from the classical targeted metabolomics to the hypothesis-generating global metabolomics approach, we are able to address the need for novel diagnostic metabolites for PDE-ALDH7A1 and provide deeper insights into the biochemical effects of ALDH7A1 deficiency [10,16].

The aim of this study was to understand the biochemistry of PDE-ALDH7A1 and discover new biomarkers in plasma samples from PDE-ALDH7A1 patients using global metabolomics. In addition, the interdependence of these metabolites as well as the stability and conversion of HACA were investigated. The present study also examined the possible influence of pyridoxine levels on the measurement of the diagnostic markers that were discovered.

## 2. Results

### 2.1. Two New PDE-ALDH7A1 Biomarkers

**Figure 1:**
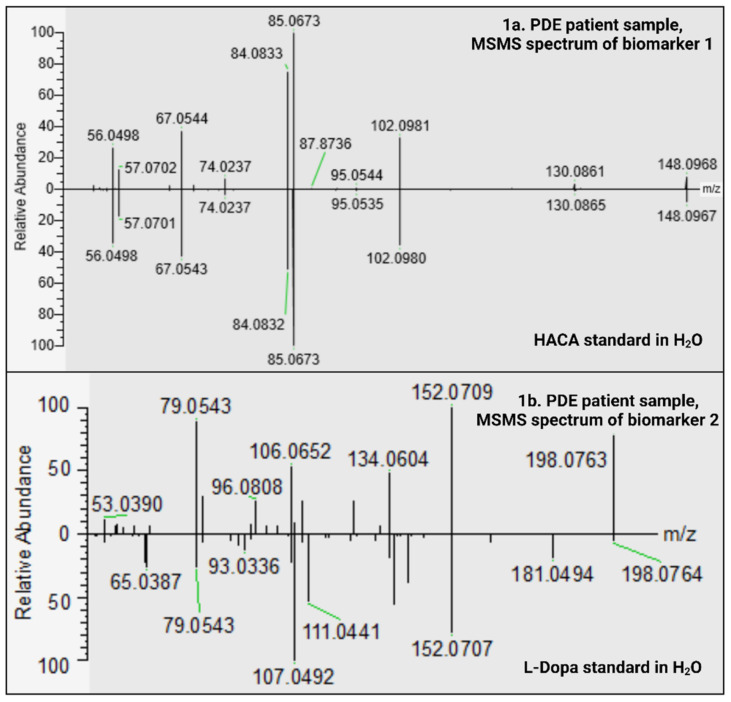
Mirror plots of observed MSMS spectra for biomarker 1 in a patient sample vs. an HACA standard in H_2_O (1a) as well as biomarker 2 in a patient sample vs. an L-dopa standard in H_2_O (1b). The intensity is arbitrary and was set to a value of 100. (1a) The *m*/*z* 148.0967 is the detected *m*/*z* of the molecular ion [C_6_H_13_NO_3_+H]^+^, whereas *m*/*z* 85.0673 represents the base peak. All peaks aligned, and no fragment had a mass deviation above 5 ppm. (1b) The *m*/*z* 198.0764 is the detected *m*/*z* of the molecular ion [C_9_H_11_NO_4_+H]^+^. Some peaks aligned with a mass deviation below 5 ppm, but the majority did not. Of note: The RT of C_9_H_11_NO_4_ was 6.412 min, whereas the RT of L-dopa was 3.498.

**Figure 2:**
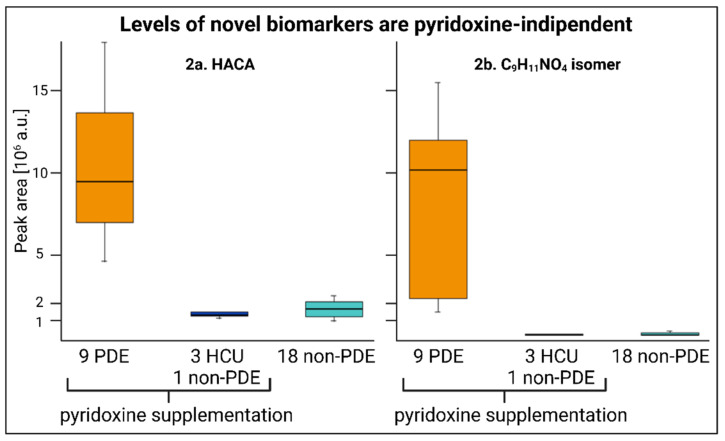
Box-and-whisker plot showing peak area comparison of HACA (**left**) and C_9_H_11_NO_4_ (**right**) in 31 plasma samples. The peak area was obtained from PDE-ALDH7A1, HCU, and non-PDE-ALDH7A1 samples (one non-PDE-ALDH7A1 patient was receiving pyridoxine supplementation). *p*-values between PDE and non-PDE groups: 0.0203 and 0.0003 for C_9_H_11_NO_4_ isomer and HACA, respectively (3 HCU and 1 non-PDE vs. 9 PDE); 0.0212 and 0.0004 for C_9_H_11_NO_4_ isomer and HACA, respectively (18 non-PDE vs. 9 PDE).

**Figure 3:**
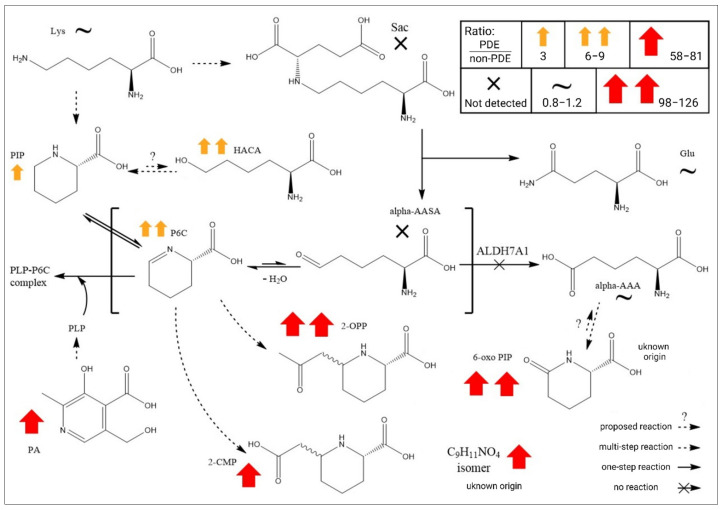
Simplified state-of-the-art PDE-ALDH7A1 metabolite profile in plasma. The ratio for each metabolite was calculated by dividing its median peak area in PDE-ALDH7A1 by the median peak area detected in non-PDE-ALDH7A1. Abbreviations: P6C (1-piperideine-6-carboxylic acid), alpha-AASA (alpha-aminoadipic semialdehyde), alpha-AAA (alpha-aminoadipic acid), 2-OPP ((2S, 6S/R)-6-(2-oxopropyl)-piperidine carboxylic acid), 2-CMP ((2S, 6S/R)-6-(2-carboxymethyl)-piperidine-2-carboxylic acid), 6-oxo PIP (6-oxo-pipecolinic acid), HACA ((S)-2-amino-6-hydroxyhexanoic acid). All metabolites were identified at level 1, except 2-CMP (level 2) and the C_9_H_11_NO_4_ isomer (level 3). Of note: only traces of 6-oxo PIP, 2-OPP, and the C_9_H_11_NO_4_ isomer were detected in non-PDE-ALDH7A1 samples. Therefore, the obtained ratio is much higher. Created with ChemDraw (ChemDraw—PerkinElmer Informatics, accessed on 14 December 2022).

Differences in the metabolomes between the PDE-ALDH7A1 and non-PDE-ALDH7A1 plasma samples were investigated using our in-house metabolomics analysis method. The PCA plots revealed tight clustering of the PQC samples, indicating good data quality. However, there was no separation between PDE-ALDH7A1 and not-PDE-ALDH7A1 in the PCA-plots. When studying the signals of known biomarkers, such as 6-oxo PIP, the two 2-OPP isomers, 2-CMP, PIP, and P6C, they were easily identified and were significantly increased in PDE-ALDH7A1 compared to non-PDE-ALDH7A1 (Figure 1, Figure 2 and Figure 3). The most interesting result emerging from the data was the discovery of two significantly increased metabolites that were not previously detected in PDE-ALDH7A1 patients (Table 1).

#### 2.1.1. HACA; Level 1 Identification of Biomarker 1

Both our preliminary studies, as well as our recent publication [2], suggested HACA as a potential biomarker for PDE-ALDH7A1 (Table 1). In order to confirm our hypothesis, an HACA standard was analyzed in different experimental setups, as presented in Figure 1a and Supplementary Figure S1(a vs. b and c). The most striking observation was that biomarker 1 shared the same retention time and MSMS spectrum as the HACA standard. We therefore refer to biomarker 1 when writing HACA in this paper.

#### 2.1.2. C_9_H_11_NO_4_; Level 3 Identification of Biomarker 2

Biomarker 2, with the formula C_9_H_11_NO_4_, was suggested to be L-dopa by CompoundDiscoverer. However, the L-dopa standard in our in-house library eluted ~3 min earlier (RT of L-dopa: 3.498 min) than biomarker 2 (RT: 6.412 min), demonstrating that biomarker 2 could not be L-dopa. Furthermore, as shown in Figure 1b, the MSMS spectrum of the L-dopa standard differed from the biomarker’s spectrum.

In Figure 1b (and Figure S2), a possible fragmentation pattern for the C_9_H_11_NO_4_ isomer (biomarker 2) is depicted. Firstly, the neutral loss of HCOOH leads to the base peak. Secondly, the initial loss of CO followed by either H_2_O (leading to *m*/*z* 106.0652) or NH_3_ and CO (leading to *m*/*z* 79.0543) leaves two fragments to dominate the MSMS spectrum. The base peak is also able to lose H_2_O directly, resulting in another significant peak in the spectrum of the new biomarker. Using CANOPUS, a function in SIRIUS 5 for analyzing the MSMS spectrum, the C_9_H_11_NO_4_ isomer was linked to an L-alpha-amino acid.

Interestingly, we observed a similar trend in the peak area of the C_9_H_11_NO_4_ isomer and the established biomarker 2-CMP in all samples, suggesting some associations. However, the RT of C_9_H_11_NO_4_ (6.526 min) differed by 2 min from the RT of 2-CMP (4.375 min). In addition, the MSMS spectra were significantly different from each other and showed almost no similar fragments.

### 2.2. Pyridoxine-Independent Elevations

The principal pharmacological treatment of patients suffering from PDE or other pyridoxine-responsive diseases is pyridoxine at a dose of up to 500 mg daily, compared to the daily recommendation for the general public of 1–2 mg [17]. Figure 2 demonstrates that a high intake of pyridoxine did not affect the peak area for any of the new biomarkers. HACA was present in both the samples and controls but was elevated in the samples, whereas C_9_H_11_NO_4_ was only found in PDE-ALDH7A1-positive samples at low concentrations.

### 2.3. Global Metabolomics Unfolds the Biochemistry of PDE-ALDH7A1

In practice, global metabolomics detects hundreds to thousands of metabolites in a sample. In the case of PDE-ALDH7A1, only a few are clinically considered for diagnostics or monitoring purposes. Therefore, their median values were compared to one another to gain biochemical and metabolic insights. Figure 3 shows the ratios for each metabolite after setting the median peak area for PDE-ALDH7A1 as the counter and that for non-PDE-ALDH7A1 as the denominator. Interestingly, our two novel biomarkers showed increases in the same order of magnitude as those of the well-established biomarkers. PIP, HACA, P6C, 2-OPP, 2-CMP, the C_9_H_11_NO_4_ isomer, and 6-oxo PIP were significantly increased in the PDE-ALDH7A1 samples. There were no significant changes regarding 6-oxo PIP, 2-OPP, HACA, the C9H11NO4 isomer, P6C, 2-CMP, pyridoxic acid, PIP, or lysine in the samples of the PDE-ALDH7A1 patients on a lysine-restricted diet, and there were no significant changes in the signals of lysine, glutamine, and alpha-AAA in the PDE-ALDH7A1 samples. Pyridoxic acid was significantly increased in the PDE-ALDH7A1 samples, in line with the high intake of pyridoxine.

### 2.4. Stability Test and Conversion of HACA

To assess the stability of HACA, the peak area of HACA in a PDE-positive control sample was measured over time (Figure S3). HACA presented robust signals by yielding a high peak area from a 1.5-year-old sample stored at −20 °C. Additionally, there was no significant difference in the signal response (98%) when the same sample was reanalyzed after 15 days of storage in the instrument autosampler at 4 °C (measurement no. 2). After 32 days (measurement no. 4), the peak area of HACA was reduced to one third (32%) of the initial value. Generally, there was a clear trend of a decreasing HACA peak area over time. After 39 days (4 °C), the peak area was 17% of the initial value.

Our results indicated an association between HACA and PIP. Therefore, the levels of both were measured in a dilution series of HACA in distilled water (Figure S4). Strikingly, there was a high degree of linearity between the concentrations of added HACA and the peak area response of PIP at equilibrium. Importantly, the MSMS spectra of PIP in the PDE-ALDH7A1 patient samples, HACA, and the PIP standard were identical, meaning that PIP is verified independently in a PDE-ALDH7A1 sample and in the HACA standard.

## 3. Discussion

This study demonstrated the powers of global metabolomics in biomarker discovery and validation for PDE-ALDH7A1. Two novel diagnostic markers, HACA and the C_9_H_11_NO_4_ isomer, were identified in a pyridoxine-independent manner. The same single-run analysis detected accumulation of all the other known PDE-ALDH7A1 biomarkers: 6-oxo PIP, 2-OPP, 2-CMP, P6C, and PIP [2,18]. Explanations for why the two novel biomarkers presented here had not been discovered previously may be related to widely varied methodology and instrumental parameters and the lack of sufficient interlaboratory standardization [19].

**Figure 6:**
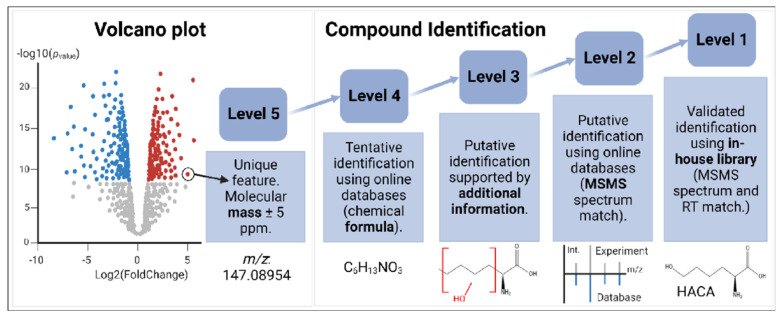
The levels of confidence in metabolite identification [43]. The volcano plot compares two groups to find significantly altered features in the metabolome at level 5. The requirements for reaching higher levels of confidence in compound identification are displayed. Abbreviation: RT (retention time). Created with BioRender.com, accessed on 14 December 2022.

Metabolite identification (levels 1–5) has been a major bottleneck in MS-based metabolomics studies [20,21]. In the current work, HACA was initially identified at level 5 (Figure 6) after comparing data from PDE-ALDH7A1 patients with the control groups (non-PDE-ALDH7A1 and/or not-PDE-ALDH7A1). The resulting low *p*-value of 3.9 E-07 from 9 PDE-ALDH7A1 (rare IEM), 19 non-PDE-ALDH7A1 (similar clinic to PDE-ALDH7A1), and 3 HCU (similar pyridoxine intake to PDE-ALDH7A1) individuals consolidated the significance of these findings. Additionally, the sample from a PDE-ALDH7A1 patient spiked with an HACA standard showed an increase of the peak area (Figure S1). Moreover, an alignment was observed after comparing the MSMS spectrum of HACA in a PDE-ALDH7A1 patient sample with the corresponding aqueous standard (Figure 1a). The fact that the mass deviation of the important fragments in both spectra did not exceed 5 ppm strengthened the power of our discovery. The C_9_H_11_NO_4_ isomer, however, was identified at level 3 (Figure 6). The molecule mostly resembled a L-alpha-amino acid [22] with the same mass and formula as D/L-dopa but with a different structure. Its delayed RT (6.412 min) when compared to L-dopa (3.498 min) might imply a more hydrophobic property, as in our method (reversed-phase chromatography) the elution time is positively correlated with hydrophobicity. From such physicochemical parameters, we conceivably hypothesize that the molecule could be an isomer of amino(4-hydroxy-3-methoxyphenyl)acetic acid (CAS: 56246-88-9). Our postulation is further supported by the main fragmentation pattern in Figure 1 and Figure S2 [23].

**Figure 4:**
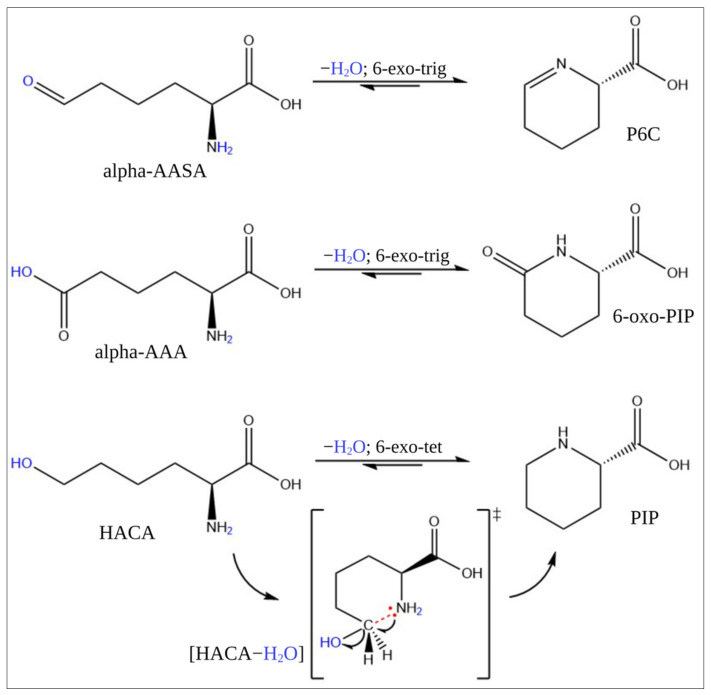
Proposed ring closure reactions of alpha-AASA, alpha-AAA, and HACA, including the transition state for HACA to PIP. The blue font and the red part are meant for visualization only. Note: all cyclization reactions shown here are favorable according to Baldwin’s rules. Abbreviations: P6C (1-piperideine-6-carboxylic acid), alpha-AASA (alpha-aminoadipic semialdehyde), alpha-AAA (alpha-aminoadipic acid), 6-oxo PIP (6-oxo-pipecolinic acid), HACA ((S)-2-amino-6-hydroxyhexanoic acid). Created with ChemDraw (ChemDraw—PerkinElmer Informatics, accessed on 14 December 2022).

On the question of metabolic conversion, this study found that HACA is most likely in equilibrium with PIP through a 6-exo-tet ring closure reaction, according to the Baldwin rules of cyclization (Figure 4). The essence of the rules is that all intramolecular exo-tet and exo-trig cyclizations are favorable [24]. Analyzing the HACA standard revealed that HACA and PIP are linearly correlated in MilliQ water (without enzymes) at 4 °C. Interestingly, the [HACA−H_2_O+H]^+^ ion (Figure S4), a possible intermediate between HACA and PIP, with a similar RT to [HACA+H]^+^ and presumably formed by losing a water molecule from HACA, was also present, indicating that HACA, already under soft ionization methods, would readily lose a water molecule. The similar RTs between [HACA+H]^+^ and [HACA-H_2_O+H]^+^ convinced us that the ion was formed upon electrospray ionization when entering the MS. However, the similar RTs of PIP from our in-house library and PIP formed in MilliQ water with only HACA, as well as the identical MSMS spectra of PIP (in-house library) and PIP formed in MilliQ water with only HACA, led us to hypothesize an enzyme-independent equilibrium between HACA and PIP, as described in the Baldwin rules.

Two other known metabolites in PDE-ALDH7A1, alpha-AASA and P6C, are in equilibrium [6,25]. However, the underlying mechanism is still not fully understood. This might also be explained by the Baldwin rules of cyclization [24,26], where the ring closure of alpha-AASA is favored by the 6-exo-trig reaction instead of opening (Figure 4). The tendency towards the formation of a ring structure (i.e., P6C) relies on the non-enzymatic reaction taking place by the electrophilic property of carbon in the aldehyde group and the ability of hydroxide/water as an excellent leaving group.

Regarding 6-oxo PIP, it is known to be the cyclic lactam of alpha-AAA [27]. The direct enzymatic formation of 6-oxo PIP from alpha-AAA has previously been reported in *Penicillium chrysogenum* [28]. *Streptomyces clavuligerus* and other cephamycin-D-producing actinomycetes possessing P6C-carboxylate dehydrogenase have implied the gut as a potential origin of 6-oxo PIP [29]. Later, the compound was found in the fecal metabolome [30]. For urine, however, a different pattern has been observed. For example, a GC-MS-based metabolomics study on samples from PDE-ALDH7A1 patients could not detect any increase in the level of alpha-AAA, in contrast to elevated 6-oxo PIP [18]. In our routine laboratory analyses, we detected 6-oxo PIP in urine from non-PDE-ALDH7A1 patients. However, compared to typical PDE-ALDH7A1, the levels were, as expected, much lower than in the PDE-ALDH7A1 samples, and 6-oxo PIP was not detected in the plasma of non-PDE-ALDH7A1. This indicates a multi-source origin of 6-oxo PIP, both dependent and independent of a defect in the *ALDH7A1* gene, which can eventually be transformed to alpha-AAA through 6-exo-trig cyclization (Figure 4). This turns 6-oxo PIP into a potential precursor for the surprisingly similar alpha-AAA levels detected in PDE-ALDH7A1 compared to control, despite being behind the enzymatic block. This may explain the absence of 6-oxo PIP in incubated P6C with acetoacetate in different matrices [10]. Instead, 2-OPP, a P6C-related metabolite, was increasingly formed under defined physiological conditions. This also accords with our MSMS spectral analysis of P6C and P6C-related metabolites such as 2-OPP, 2-CMP, and PIP. These spectra showed a clear trend of the MSMS spectra consisting of similar fragments, indicating similar origins. It is noteworthy that the MSMS spectrum of 6-oxo PIP differed strongly from the MSMS spectrum of P6C, PIP, 2-OPP, and 2-CMP, supporting the idea of a different root than these P6C-related metabolites, whereas one can find the same, most intense fragments in the MSMS spectra of alpha-AAA and 6-oxo PIP, strengthening their possible biochemical relation.

Another interesting question is the mechanism of formation of the two biomarkers identified in the present study. Uncertainty in the identification of the final metabolite for the C_9_H_11_NO_4_ isomer (level 3) makes it hard to point in a particular direction. HACA, at level 1 of identification confidence, was linked to oxidative stress in a recent review from our group [2]. The occurrence of oxidative stress is known for *ALDH7A1* deficiency [29,31,32], and HACA has been shown to be a highly specific marker of metal-catalyzed protein oxidation [33,34]. More research is needed to identify the C_9_H_11_NO_4_ isomer at a higher level of confidence and to better understand the formation mechanisms of both biomarkers.

We also observed HACA’s six-fold increase in heparin plasma samples of PDE-ALDH7A1 patients when compared to not-PDE-ALDH7A1s. A final question that needs to be answered is the potential role of HACA. One study demonstrated its antagonistic nature to lysine, competitively inhibiting the incorporation of this essential amino acid in protein synthesis [35]. This may imply that HACA could be able to interfere in lysine metabolism. A possible consequence of this would be increased levels of lysine available for catabolism, thus augmenting the levels of other neurotoxic metabolites. Elevated levels of alpha-AASA, P6C, and pipecolic acid are believed to be neurotoxic, which may impair neurodevelopmental outcomes [36,37]. Despite early diagnosis and adequate seizure control, the vast majority of PDE-ALDH7A1 patients suffer from intellectual disabilities or developmental delays [38]. Hence, lysine reduction therapies, consisting of restricted lysine intake (less substrate) and arginine supplementation (a competitive inhibitor of lysine transport across the intestinal epithelium and blood–brain barrier) as well as pharmacological doses of pyridoxine are the recommended treatment [3]. This strategy has been shown to be effective in terms of reduced accumulations of alpha-AASA, P6C, and pipecolic acid as well as improvements in both cognitive development and seizure management [7,39]. However, more research on this topic needs to be undertaken before the association between HACA and lysine is fully understood.

## 4. Material and Methods

### 4.1. Reagents

Formic acid (98%) and water of type 1 (>18 MΩ cm) from a MilliQ ultrapure water purification system were from Merck Millipore (Darmstadt, Germany). Liquid chromatography–mass spectrometry (LC–MS)-grade methanol was purchased from Rathburn Chemicals (Walkerburn, Scotland). The remaining reagents were: 6-Hydroxy-2-aminocaproic acid (HACA) (Alichem Inc. (San Diego, CA, USA)), L-2-Aminohexanedioic acid (aAASA) (Toronto Research Chemicals (Toronto, ON, Canada)), and pipecolic acid (Sigma-Aldrich (Darmstadt, Germany)).

### 4.2. Patients and Clinical Details

Four male and five female patients between the ages of 11 months and 31 years (median: 16.5 years) with genetically confirmed PDE-ALDH7A1 were included in this study. All nine presented with neonatal seizures resistant to conventional antiseizure medications, and all received pyridoxine treatment from the neonatal period. The pyridoxine doses at the time of sampling ranged from 40 to 500 mg/day (median: 252 mg). The five youngest patients (aged 11 months to 16 years) also received a lysine-restricted and arginine-fortified diet. All controls were carefully matched with regard to gender and age. The non-PDE control group consisted of 11 male and 8 female subjects between the ages of 2 months and 41 years (median: 11.9 years). The HCU control group consisted of one male (age: 4.5 years) and two females (ages: 33 and 44).

### 4.3. Ethics Statement

The study was approved by the Regional Committee for Medical and Health Research Ethics (case No.: 200284).

### 4.4. Experimental Procedure

#### 4.4.1. Patients, Controls, and Samples

Heparin-anticoagulated plasma samples from the 9 patients with PDE-ALDH7A1 and 22 controls were analyzed. For the latter study group, 19 individuals (non-PDE) had previously been through a diagnostic evaluation for seizures but without pathological findings of PDE-ALDH7A1 biomarkers. The remaining three controls had homocystinuria (HCU) and received therapeutic doses of pyridoxine equivalent to those of the nine PDE patients. All samples were stored at −20 °C prior to analysis for a period ranging from a few days to two years.

#### 4.4.2. Sample Preparations and UHPLC-HRMS Analysis

**Figure 5:**
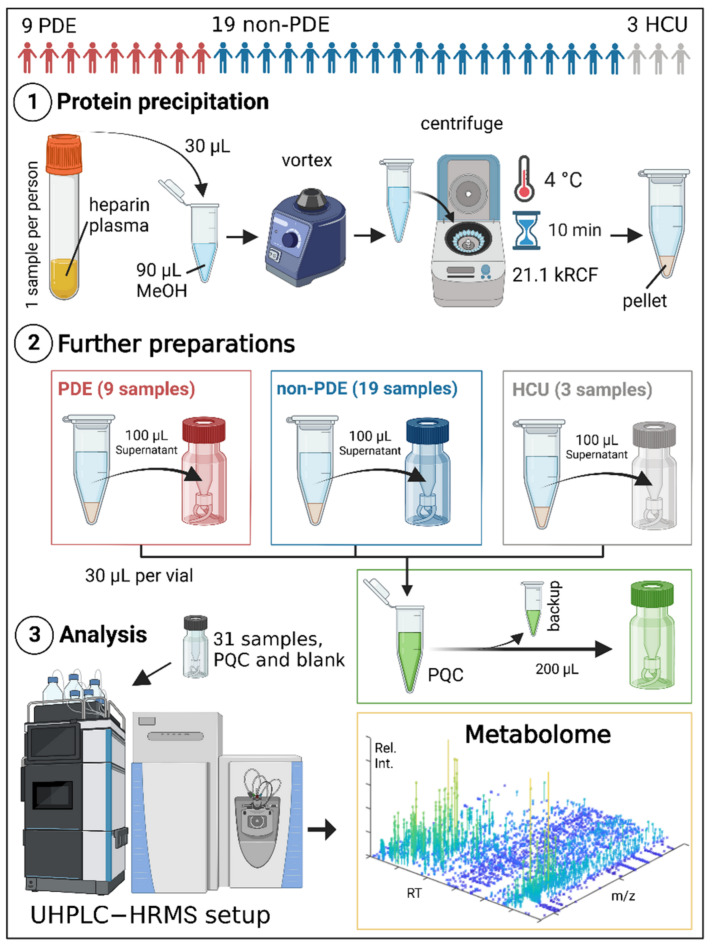
Flow chart describing the process of obtaining the metabolome in this study. After protein precipitation, 30 µL of the 100 µL of plasma was collected from each vial to make the pooled quality control (PQC) sample. The PQC sample was used for analytical correction, and a processed blank was used for background extraction. Abbreviations: UHPLC (ultra high performance liquid chromatography), HRMS (high-resolution mass spectrometry). Created with BioRender.com, accessed on 14 December 2022.

The average number of freeze–thaw cycles was three for most samples (range: 1–4). All plasma samples were thawed, vortex-mixed (Reax top vortex mixer, Heidolph (Schwabach, Germany)), and centrifuged (Megafuge 1.0R, Heraeus (Hanau, Germany)) for 10 min at 3600 RCF (Figure 5). The samples were then mixed with 4 °C cold methanol (1:3 sample/methanol, *v*/*v*), vortex-mixed, and centrifuged (Fresco 21 Microcentrifuge, Thermo Scientific (Waltham, MA, USA)) for 10 min (4 °C) at 21,100 RCF. The supernatant was then transferred into a sample vial from which it was injected into the LC-MS system.

In order to evaluate the overall quality of the metabolomics data, a principal component analysis (PCA) was carried out. The pooled quality control (PQC) samples were tightly clustered, certifying the quality of the global metabolomics data that were obtained. The PQC samples were prepared by mixing 30 µL from all samples and were analyzed at the start, the end, and after every fifth study sample for signal correction within the whole analysis. Three pooled metabolite identification (PMI) samples were prepared by collecting 30 µL from each of the 9 PDE-ALDH7A1, 19 non-PDE-ALDH7A1, and 3 HCU samples. This was carried out to perform an MSMS analysis for metabolite identification purposes. Furthermore, a processed blank sample consisting of 30 µL of water and 90 µL of methanol, treated the same way as the other samples before analysis, was included in the analysis to correct for background signals.

To test the stability of our proposed biomarkers, we used a positive control consisting of pooled samples from two confirmed cases of PDE-ALDH7A1 that had been aliquoted and stored at −20 °C for 1.5 years prior to the analysis. One of the aliquots was transferred to the autosampler of the UHPLC instrument, kept at 4 °C, and analyzed at three time points with two-week intervals.

The UHPLC instrumentation was a Dionex Ultimate 3000 UHPLC system pump, a column department, and an autosampler; all from Thermo Scientific (Bremen, Germany). The MS was a Q Exactive Orbitrap, also from Thermo Scientific, with electrospray ionization (ESI) operated in the positive-ionization mode. A full MS analysis, including liquid chromatography and electrospray ionization parameters and settings, was performed in the positive-ionization mode according to the in-house procedure described by Skogvold et al. [40]. The settings used for all MSMS analyses performed with the Q Exactive Orbitrap can be seen in Table S1 (Supplementary Materials). The peak area ratios were calculated by dividing the median of PDE-ALDH7A1 by non-PDE-ALDH7A1.

The experiments were carried out three times by two operators at the National Unit for Screening and Diagnosis of Congenital Pediatric Metabolic Disorders, the Department of Medical Biochemistry, Oslo University Hospital (Rikshospitalet), Norway. The laboratory participates in several external quality programs: LabQuality and the European Research Network for Evaluation and Improvement of Screening, Diagnosis and Treatment of Inherited Metabolic Disorders (ERNDIM).

#### 4.4.3. Computer Software

Xcalibur SP1 (version 4.2.47, Xcalibur™ Software (thermofisher.com, accessed on 14 December 2022)), Compound Discoverer (version 3.1, Compound Discoverer Software | Thermo Fisher Scientific - NO), and FreeStyle (version 1.6, Freestyle Software Support Getting Started | Thermo Fisher Scientific - NO) from Thermo Scientific (Bremen, Germany) were used for obtaining, processing, and visualizing the metabolomics data, respectively. The workflow “WorkflowTemplates\LC\Metabolomics\Untargeted Metabolomics with Statistics Detect Unknowns with ID using Online Databases and mzLogic” was used for data processing. BioRender (San Francisco, CA, USA) was used for illustrations. SIRIUS (version 5.5.4; Uni-Jena) [41] and CSI:FingerID (Böcker Group, Uni-Jena) [42] were used to achieve higher levels of confidence in metabolite identification.

### 4.5. Levels of Confidence in Compound Identification

Figure 5 illustrates the overview of our metabolomics procedure. The collected metabolome can be used in different ways, ultimately leading to a better understanding of diseases, with subsequently improved diagnostics and therapeutic monitoring. For the purpose of diagnosing PDE-ALDH7A1 in biological samples (plasma, urine, and cerebrospinal fluid), we extracted the protonated molecular mass [M+H]^+^ ± 5 ppm of 6-oxo PIP and 2-OPP from the metabolome. For more insights into the biochemistry, we also extracted P6C, alpha-AASA, PIP, and 2-CMP from the already acquired metabolome. Since the metabolome is a dataset, this could be achieved simultaneously.

The levels of confidence of identification ranged from 1 to 5 [43], where 1 was the highest level (Figure 6). In our study, upon metabolome collection, significant group-specific metabolite signatures were found using volcano plots. The criteria for this comparison of molecular features between groups were *p*-value < 0.05 and log2 fold change ±1. To reach level 1 of identification, we used our in-house library containing the exact masses of molecular ions, fragmentation spectra, and retention times (RTs) of several hundred reference compounds analyzed on our platform. To reach level 2 and below, we used both SIRIUS and Compound Discoverer. The latter used Chemspider for the proposal of molecular formulae based on MS1 spectra, especially isotopic patterns, in order to search for matches in online databases. In addition, mzCloud used online fragmentation patterns to increase the confidence in the proposed molecular identity.

## 5. Conclusions

This study demonstrated the discovery of two novel biomarkers, HACA and an isomer of C_9_H_11_NO_4_, in PDE-ALDH7A1 plasma samples by UHPLC-HRMS-based global metabolomics. In addition, a possible reaction mechanism for one of the biomarkers, HACA, was proposed. We also demonstrated how global metabolomics, in combination with the monitoring of all known biomarkers, could be applied as a routine diagnostic tool for identifying not only PDE-ALDH7A1 but virtually any disease.

## Figures and Tables

**Table 1:** Results for biomarkers obtained from global metabolomics, confirmed in 9 PDE-ALDH7A1 patients compared with 19 non-PDE-ALDH7A1 control patients.

Feature	*m*/*z*	RT (min)	Molecular Formula (Compound Discoverer)	Fold Change (Sample/Control)	*p*-Value
Biomarker 1	148.09668	2.442	C_6_H_13_NO_3_	5.852	3.9 E-7
Biomarker 2	198.07602	6.526	C_9_H_11_NO_4_	84.162	1.6 E-6

## Supplementary Materials

The following supporting information can be downloaded at: https://www.mdpi.com/article/10.3390/ijms232416061/s1.
