# Supplementary material for: Global Metabolomics Discovers Two Novel Biomarkers in Pyridoxine-Dependent Epilepsy Caused by ALDH7A1 Deficiency
Source: Int J Mol Sci. 2022 Dec 16;23(24):16061. doi: 10.3390/ijms232416061 (PMC9784804; doi:10.3390/ijms232416061)
Supplement: Supplementary file 1 [file ijms-23-16061-s001.zip › ijms-2059630-supplementary.pdf]

Supplementary Figures and Table

Figure S1. Representative chromatogram of $[HACA+H]^+$ from a sample from a confirmed PDE-ALDH7A1-patient not spiked (S1A), spiked with 1 µM (S1b) and spiked with 100 µM HACA (S1c). The retention time of HACA is 2.442 min. Automatic area (AA) refers to the automatically integrated area under the curve.

Figure S2. Fragmentation tree [1] obtained from SIRIUS 5 software describing the fragmentation pattern of the $C_9H_{11}NO_4$ isomer. The descending presentation of molecular formula in boxes reflects fragments lost as neutral molecules. The relative intensity from the MSMS spectrum in Figure 5 is given in both color intensity and number.

Figure S3. Yielded relative peak area of HACA from a prepared sample which has been stored for 1.5 years at -20°C. As from the first analysis, this internal positive control has been kept in the autosampler at 4 °C and the peak area has been normalized to the day 0 run.

Figure S4. Left: Representative extracted ion chromatogram of the masses of PIP (top-left) and HACA (bottom-left). PIP is detected after adding HACA into water. Visualization of the possible conversion between HACA and PIP via the $[HACA-H_2O+H]^+$-ion. Right: Peak area value of PIP in dependence of amount HACA injected. Linear regression of the dataset leads to the function $f(x) = 2E+06 \cdot x + 590919$.

Table S1. Settings used for all MSMS analyses performed with the Q Exactive Orbitrap.

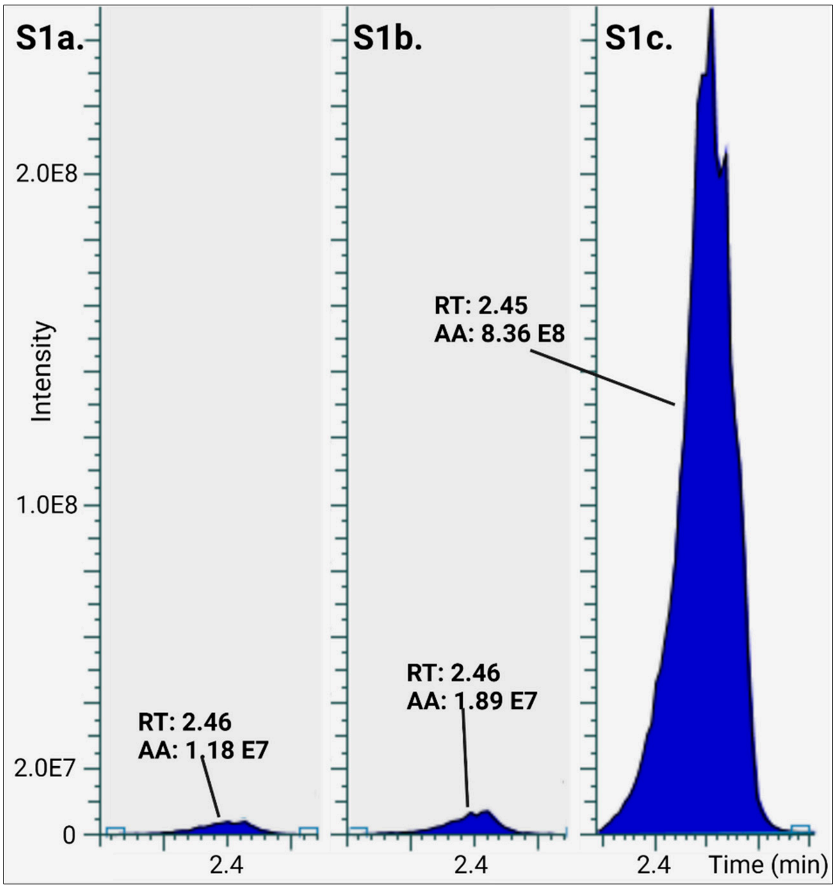
S1a.
S1b.
S1c.
2.0E8
1.0E8
2.0E7
0
Intensity
RT: 2.46
AA: 1.18 E7
RT: 2.46
AA: 1.89 E7
RT: 2.45
AA: 8.36 E8
2.4
2.4
2.4
Time (min)

Figure S1.

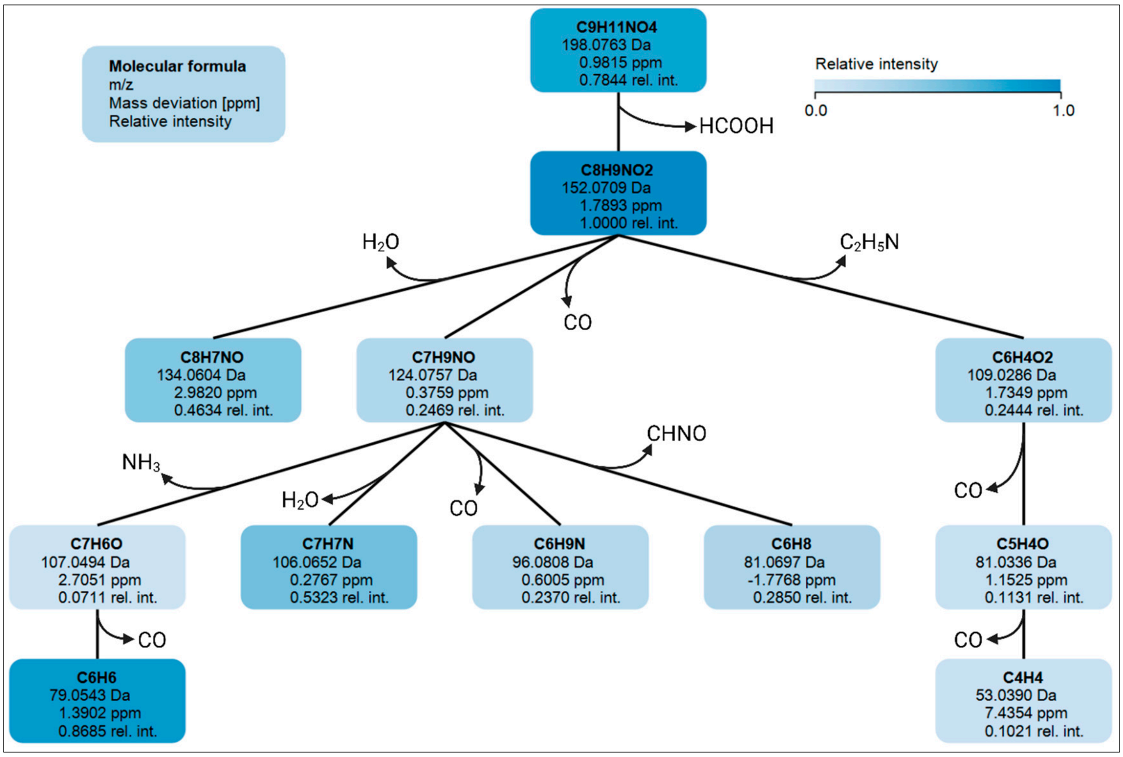

Molecular formula
m/z
Mass deviation [ppm]
Relative intensity
Relative intensity
0.0
1.0
C9H11NO4
198.0763 Da
0.9815 ppm
0.7844 rel. int.
HCOOH
C8H9NO2
152.0709 Da
1.7893 ppm
1.0000 rel. int.
H2O
CO
C2H5N
C8H7NO
134.0604 Da
2.9820 ppm
0.4634 rel. int.
C7H9NO
124.0757 Da
0.3759 ppm
0.2469 rel. int.
C6H4O2
109.0286 Da
1.7349 ppm
0.2444 rel. int.
NH3
H2O
CO
CHNO
CO
C7H6O
107.0494 Da
2.7051 ppm
0.0711 rel. int.
C7H7N
106.0652 Da
0.2767 ppm
0.5323 rel. int.
C6H9N
96.0808 Da
0.6005 ppm
0.2370 rel. int.
C6H8
81.0697 Da
-1.7768 ppm
0.2850 rel. int.
C5H4O
81.0336 Da
1.1525 ppm
0.1131 rel. int.
CO
CO
C6H6
79.0543 Da
1.3902 ppm
0.8685 rel. int.
C4H4
53.0390 Da
7.4354 ppm
0.1021 rel. int.

Figure S2.

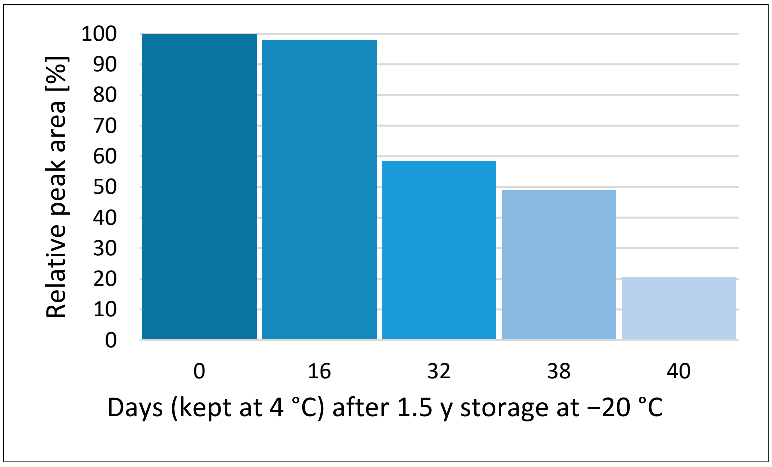
Relative peak area [%]
100
90
80
70
60
50
40
30
20
10
0
0
16
32
38
40
Days (kept at 4 °C) after 1.5 y storage at −20 °C

Figure S3.

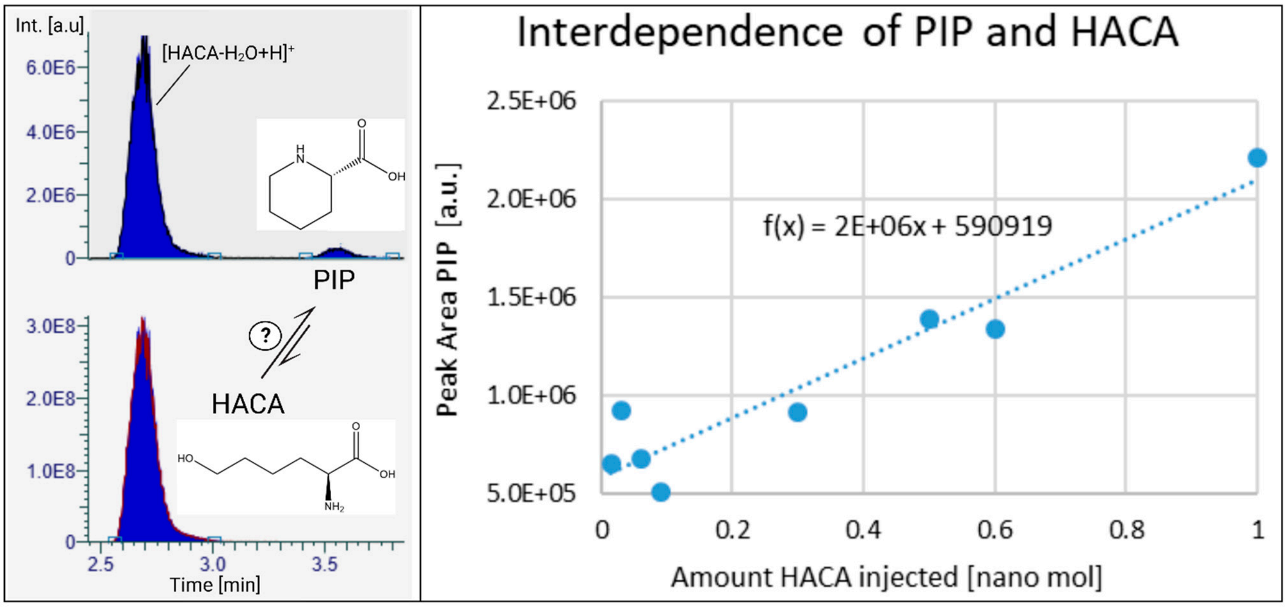
Int. [a.u]
6.0E6
4.0E6
2.0E6
0
[HACA-H2O+H]+
PIP
?
HACA
3.0E8
2.0E8
1.0E8
0
2.5
3.0
3.5
Time [min]
HO
OH
NH2
Interdependence of PIP and HACA
Peak Area PIP [a.u.]
2.5E+06
2.0E+06
1.5E+06
1.0E+06
5.0E+05
f(x) = 2E+06x + 590919
0
0.2
0.4
0.6
0.8
1
Amount HACA injected [nano mol]

Figure S4.

Table S1. Settings used for all MSMS analyses performed with the Q Exactive Orbitrap.

Scan range	*m/z* 50 – 750
Resolution	Full MS: 70 000
	MSMS: 17 500
AGC target value	Full MS: 1 000 000 ion counts
	MSMS: 500 000 ion counts
Maximum injection time	Full MS: 200 ms
	MSMS: 100 ms
Polarity	Positive
Scan type	Data dependent acquisition, top 5
Dynamic exclusion time	10 s
Intensity threshold	50 000 counts per second
Stepped normalized collision energy	20, 50, 80

References

1. Böcker, S.; Dührkop, K. Fragmentation trees reloaded. *Journal of cheminformatics* **2016**, *8*, 1-26.
